# Immunomodulation Driven by Theranova Filter during a Single HD Session

**DOI:** 10.3390/jcm13072147

**Published:** 2024-04-08

**Authors:** Carlotta Caprara, Grazia Maria Virzì, Katia Chieregato, Nicola Marchionna, Valentina Corradi, Alessandra Brendolan, Claudio Ronco, Monica Zanella

**Affiliations:** 1 IRRIV—International Renal Research Institute Vicenza Foundation, 36100 Vicenza, Italy; carlotta.caprara@aulss8.veneto.it (C.C.); graziamaria.virzi@aulss8.veneto.it (G.M.V.); cronco@goldnet.it (C.R.); 2 Department of Nephrology, Dialysis and Transplantation, AULSS8 Berica, Ospedale San Bortolo, 36100 Vicenza, Italy; nicola.marchionna@aulss8.veneto.it (N.M.); brendolan.alessandra@gmail.com (A.B.); monica.zanella@aulss8.veneto.it (M.Z.); 3 Advanced Cellular Therapy Laboratory, Hematology Unit, S. Bortolo Hospital, AULSS 8 Berica, Contra’ San Francesco 41, 36100 Vicenza, Italy; k.chieregato@inwind.it; 4 Hematology Project Foundation, 36100 Vicenza, Italy

**Keywords:** Theranova filter, Treg cells, HLA-DR

## Abstract

**Background**: Patients with end-stage kidney disease (ESKD) have altered immunity. Patients on hemodialysis (HD) present a coexistence of immunodeficiency and activation of the immune system. We evaluated the immunophenotypic profile induced by the medium cut-off of Theranova filter during a single HD session in the same individual. **Methods**: This pilot observational study explored 11 patients (75 ± 8 years and 73% male). Blood samples were collected prior to (predialytic, PRE) and after 4 h (postdialytic, POST) standard HD session with a medium cut-off, polyarylethersulfone and polyvinylpyrrolidone blend, BPA-free membrane. We performed an immunophenotyping characterization by using flow cytometry. We evaluated eryptosis RBCs and HLA-DR expression on monocytes and Treg cells. **Results**: The percentages of eryptosis in lymphocytes (CD3+), lymphocyte T helper (CD3+ and CD4+) cells, and monocytes (CD45+ and CD14+) were similar pre- and post-HD. On the contrary, HLA-DR expression and Treg cell numbers significantly decreased after HD. **Conclusions**: Many studies have focused on the comparison between healthy volunteers and HD patients, but very few have focused on the changes that occur after an HD session in the same individual. With this pilot observational study, we have revealed an immunomodulation driven by HD treatment with Theranova filter. Our preliminary results can be considered to be a hypothesis, generating and stimulating further studies with better designs and larger populations.

## 1. Introduction

Patients with end-stage kidney disease (ESKD) have an altered immunity compared to the general population [1].

The immune alterations seen in chronic kidney disease (CKD) and ESKD are the result of numerous influences by uremia and its treatment. Both innate and adaptative immunity are dysregulated in patients with ESKD. In particular, patients on maintenance hemodialysis (HD) present a state of immunodeficiency, leading to dysfunction in host defenses and an increased susceptibility to infections [2,3]. Paradoxically, despite the apparent manifestations of immuno-incompetence, this immunodeficiency coexists with the activation of most immunocompetent cells [4], as is revealed by markers on immune competent T-cells. Unfortunately, in this context, the roles of chronic uremia and maintenance dialysis are not successfully understood. Furthermore, substantial data have demonstrated that HD treatment induces changes in the phenotypic profile and functional state of peripheral blood mononuclear cells (PBMC) [5,6]. In addition, different groups have reported an increased basal activation state, with changes in the expression of different cell surface markers and the release of pro-inflammatory cytokines. Remarkably, these signs of activation coexist with functional defects, such as lower phagocytic activity, impaired antibody response, and reduced neutrophil chemotaxis [7,8,9,10,11].

Recently, uremia was reported to influence cytokine synthesis. Furthermore, altered eryptosis, red blood cell turnover [12], abnormal regulatory T cells functioning [13], and an imbalance in immature and memory B cells have been reported in uremia. Uremia induces a chronic inflammatory response and a state of immune deficiency, as is partially reflected by the imbalance between antigen recognition and HLA-DR expression. Monocytes with diminished or missing HLA-DR expression are strongly inhibited in their antigen presenting function and also in their ability to produce inflammatory mediators in response to a respective stimulus [14]. The above abnormalities described are further enhanced by renal replacement therapy [15].

The age-dependent loss of Treg function may evoke the controversial discussion concerning the suppressive activity of Tregs in dialysis patients, which on one hand were described as dysfunctional, but on the other hand were successfully tested for possible cell therapy after kidney transplantation. Schaier et al. [16] have reported that dialysis patients exhibited a decreasing suppressive activity with age, owing to strengthened Tresp reactivity, which could explain the higher prevalence of chronic inflammatory conditions in these patients [16].

Also, Hendrikx et al. have observed an overactivated (demonstrated through activation of gene expression markers) but functionally compromised immune system in patients with end-stage renal failure. It now appears that in this setting, regulation by CD4(+), CD25(bright+), FoxP3(+), and T-cells is also impaired [13,17].

All these data suggest that cell activation, in particular in monocytes and Treg cells, and subsequent immune dysregulation (i.e., cytokine production) are presumed to be involved in HD-related morbidity.

Many studies have compared immune cellular parameters between healthy volunteers and HD patients, but very few studies have focused on the effect of a single HD session in the same individual on T cell physiology [18].

Based on this idea, the present study was designed to analyze possible changes in immune antigen expression from HD-induced changes to blood cells. In particular, we evaluated the effect of the new medium cut-off Theranova filter on blood cells before and after HD treatment.

## 2. Materials and Methods

### 2.1. Subjects

This observational study was conducted in the hemodialysis (HD) center of the Nephrology Department at San Bortolo Hospital in Vicenza, Italy. A total of 11 patients undergoing chronic HD with the same treatment characteristics (membrane filter, surface, blood flow, dialysate type, and vascular access) were enrolled, and their medical histories were reviewed for inclusion into the study. In particular, patients included in this study were undergoing expanded hemodialysis with a medium cut-off, polyarylethersulfone and polyvinylpyrrolidone blend, BPA-free membrane (Theranova filter, Deerfield, IL, USA).

Clinical characteristics, laboratory data, and dialysis-related parameters were registered for all patients.

All procedures were performed in accordance with the Declaration of Helsinki. The protocol was approved by the Ethics Committee of San Bortolo Hospital (Del n.979, 27 May 2021, n. 25/21). Informed consent was obtained from the patients for the analyses carried out in the study.

### 2.2. Sample Collection and Laboratory Parameters

For all of the 11 patients enrolled, blood samples were collected prior to (predialytic, PRE) and after 4 h (postdialytic, POST) of a standard hemodialysis session by using Theranova filter. Blood samples were collected into ethylenediaminetetraacetic acid (EDTA)-containing tubes and processed within 1 h after venipuncture. Samples were subsequently centrifuged for 7 min at 1600× *g*.

### 2.3. Eryptosis Evaluation

RBCs were freshly isolated from all patients and samples were analyzed in parallel. Eryptosis measurements were performed by using AnnexinV. AnnexinV is used to identify eryptotic cells [19,20] that are avidly bound by phosphatidylserine (PS). Flow cytometric analyses were used to measure AnnexinV bound to PS exposed on RBC surfaces. Briefly, RBCs separated from leukocytes were washed in 5 mM CaCl2 Ringer solution (1 μL of leukocyte in 400 μL of Ringer solution) and then stained for 20 min, avoiding light, with 1 μL of AnnexinV conjugated with FITC (Beckman Coulter, Brea, CA, USA). Before analysis with Navios Flow Cytometer (Beckman Coulter, Brea, CA, USA), 400 μL of Ringer solution was added. This led to an evaluation of the subpopulations of eryptotic RBCs over 1 h. RBCs that had exposed PS at their surface were identified as gating and enumerating RBCs. After that, FSC (forward scatter) was assessed and AnnexinV fluorescent intensity was measured, with an 488 nm excitation wavelength and an 530 nm emission wavelength. A minimum of 100,000 events were collected on each sample.

### 2.4. HLA-DR Expression

HLA-DR expression on monocytes was measured by using triple color flow cytometry (Navios, Beckman Coulter, Brea, CA, USA). Additionally, for a duration of 20 min under protection from light, 100 μL of whole blood was stained by 10 μL anti-HLA-DR conjugates with allophycocyanin (APC) (Beckman Coulter, Brea, CA, USA) to determine monocytes and B-lymphocytes; 20 μL anti-CD14 conjugates with phytoerythrin (PE)(Beckman Dickinson, Biosciences, San Jose, CA, USA) to determine monocytes; and 10 μL anti-CD45 conjugates with phycoerythrin cyanin7 (PC7) (Beckman Coulter, Brea, CA, USA) to determine leukocytes. Cells were resuspended in 1X lysing solution (lysing solution 10X: 8.29 g NH_4_Cl, 1 g KHCO_3_, 0.037 g C_10_H_14_N_2_Na_2_O_8_, distilled water up to 100 mL, pH = 7.3–7.5) for 10 min. Then, 200 µL of 1X PBS (PBS 10X: 80 g NaCl, 2 g KCl, 14.4 g Na_2_HPO_4_, 2.4 g KH_2_PO_4_, pH 7.4) was added to each tube. Cells were analyzed in a cytometer equipped for multiparametric and multicolor analysis. The fsc, orthogonal side scatter (SSC), and fluorescence were determined. Cells were acquired and gated by FSC and SSC. Cells positive for CD45 and CD14 (monocytes) were isolated. A minimum 100,000 events were collected in each sample. The mean fluorescence intensity (MFI) of HLA-DR was determined by using the geometric mean value after staining with the marker antibody.

### 2.5. Treg Cells Detection

Flow cytometric analysis was performed according to standard procedures and samples were acquired on a Navios Flow Cytometer (Beckman Coulter, Brea, CA, USA). Briefly, 100 µL of whole blood was stained by 5 µL of CD3PerCP (BioLegend, San Diego, CA, USA); 5 µL of CD4FITC (BioLegend, San Diego, CA, USA); 5 µL of CD25PECy7 (BioLegend, San Diego, CA, USA); and 20 µL of CD127PE (Beckman Coulter, Brea, CA, USA).

For intranuclear FoxP3 staining, after washing with PBS 1X, cells were incubated for 2 h at room temperature with an internalization kit FIX/PERM (eBioscience, San Diego, CA, USA). The cells were washed and then incubated for 30 min at 4 °C with FOXP3 APC antibody (eBioscience, San Diego, CA, USA). Cells were then resuspended in 1X lysing solution for 10 min.

After 5 min at 400 g, the cells were resuspended in 200 µL of PBS1X in preparation for flow cytometry. At least 20,000 gated lymphocyte events were acquired from each tube. The mean fluorescence intensity (MFI) was determined by using the geometric mean value after staining with the marker antibody.

**Figure 1:**
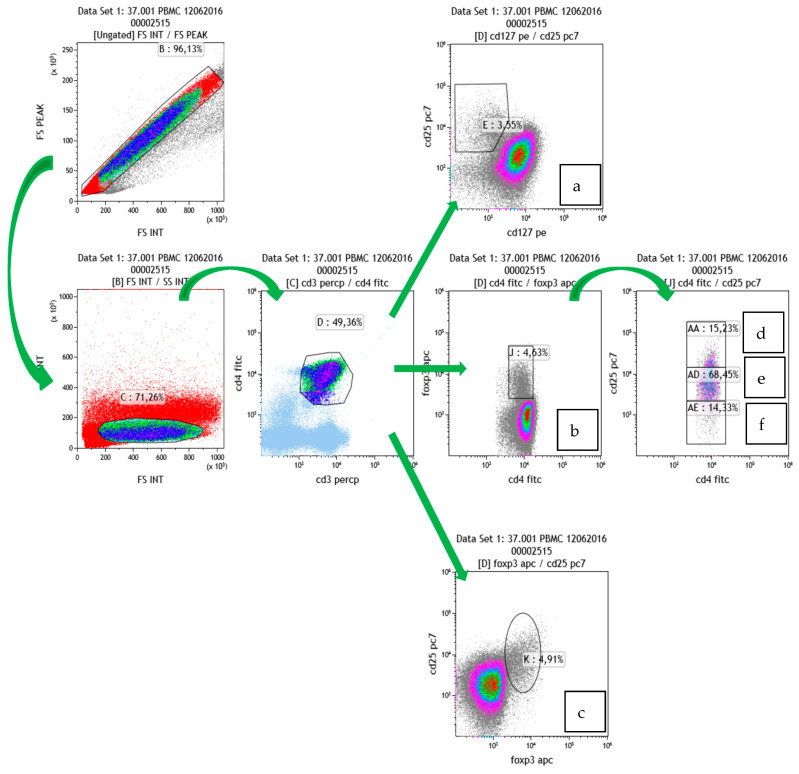
Treg gating strategy.

According to the technical paper [21], we considered CD25+ CD127- to be Treg (after selection of CD3 + CD4 + cells) (Figure 1a). To better characterize the regulatory capacity of Treg cells, we subdivided the Foxp3+ cells (of CD3 + CD4+) on their CD25+ expression. We considered the CD25hi population to be the most specific Treg population. (Figure 1d).

### 2.6. Statistical Analysis

The SPSS 16.0 (SPSS Inc., Chicago, IL, USA) software package was used to perform the statistical analysis. We used percentages to express categorical variables; means ± standard deviation (parametric variables) or median and interquartile range (IQR) (non-parametric variables) were used for continuous variables. When considered appropriate, the Mann–Whitney U test or *t*-test used were used for comparisons of two groups. Values were considered to be statistically significant if their *p*-value < 0.05.

## 3. Results

### 3.1. Subjects

The mean age of 11 HD patients was 75 ± 8 years, and 73% of these patients were male. Ten HD patients had cardiovascular disease (CVD) and six patients had diabetes. The clinical and laboratory parameters of all the HD patients are summarized in Table 1.

### 3.2. RBC Analysis and Eryptosis

In this study, we performed a sub-analysis of the HD patients to investigate immunophenotyping characteristics of the HD patients treated by using Theranova filter.

**Figure 2:**
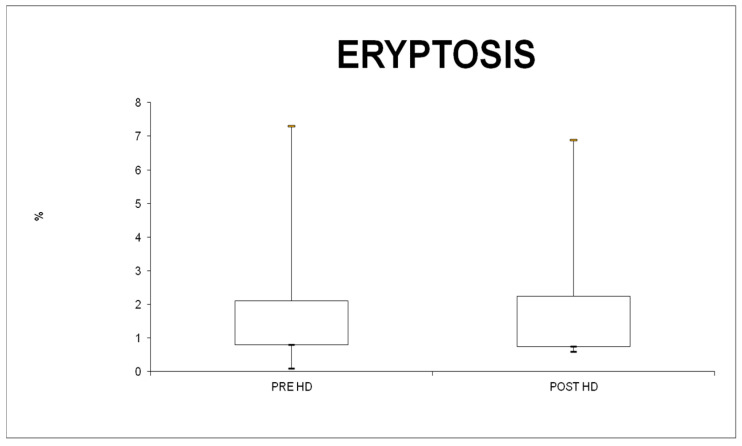
RBC analysis and eryptosis.

The percentage of AnnexinV-binding erythrocytes, reflecting the percentage of erythrocytes exposing PS at their surface, was similar in the pre- and post-HD (Theranova filter) conditions (1.0%; IQR 0.8–2.1 versus 1.0%; IQR 0.8–2.3; *p* = 0.56) (Figure 2). The average FS, which reflects cell volume, showed no significant differences in erythrocytes prior to and after the HD treatment (*p* = 0.42).

### 3.3. WBC Counts and Differentiation

The percentages of lymphocytes (CD3+), lymphocyte T helper (CD3+ and CD4+) cells, and monocytes (CD45+ and CD14+) were similar pre- and post-HD treatment with Theranova filter (*p* = 0.54, *p* = 0.42, and *p* = 0.06, respectively), as seen in Table 2.

### 3.4. Expression of Monocyte Cell Surface Marker (HLA-DR)

**Figure 3:**
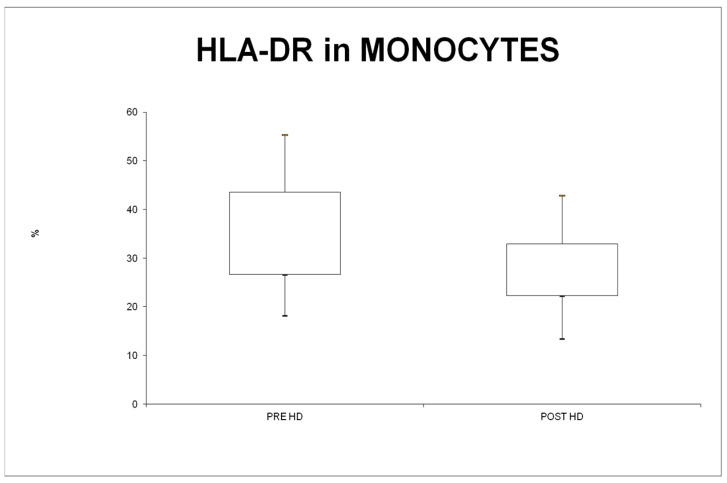
Expression of monocyte cell surface marker (HLA-DR).

After HD treatment by using Theranova filter, the expression of HLA-DR was downregulated (29.6%, IQR 26.6–43.6 versus 24.2%, IQR 22.3–33.0; *p* < 0.001 (Figure 3). On the contrary, in terms of MFI, there was no statistically significant difference between the predialytic and postdialytic levels (7.4, IQR 7.0–8.7 versus 8.4, IQR 7.2–8.7, *p* = 0.70).

### 3.5. Treg

**Figure 4:**
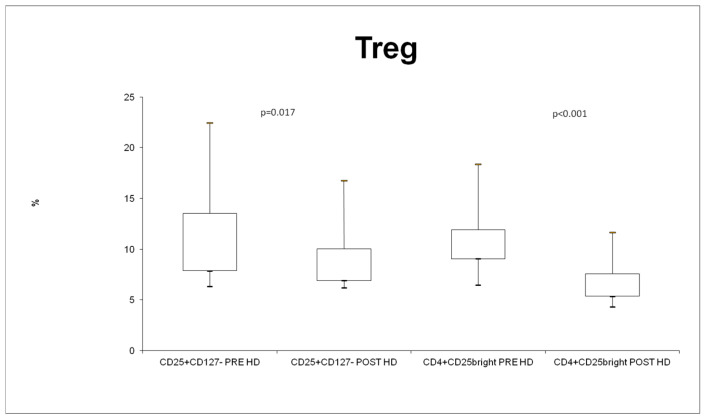
% of Treg cells evaluated in two different way.

Treg was evaluated in two different ways, as described in the materials and methods (see Figure 1) section. In both cases, Treg cells were significantly decreased after the dialysis session (Figure 4).

We considered the CD3+ CD4+ FOXP3+ population that contained Treg cells, and there was no difference before and after the treatment (median 8.86 PRE versus 9.59 POST; *p* = 0.213).

Moreover, we analyzed CD4+ CD3+ FOXP3+ CD25-medium and CD4+ CD3+ FOXP3+ CD25-low T lymphocytes, or “no Treg cells”. CD4+ CD3+ FOXP3+ CD25-medium levels significantly increased after the treatment (median 17.89 PRE versus 28.34 POST-HD; *p* = 0.001143); on the contrary, CD4+ CD3+ FOXP3+ CD25-low levels significantly decreased after the treatment (median 69.01 PRE versus 62.66 POST; *p* = 0.0146).

The expression level of FOXP3, in terms of MFI, was not statistically different between pre- and post-HD conditions (median 2.87 PRE versus 2.55 POST; *p* = 0.834).

The expression level of CD25, in terms of MFI, was not statistically different between pre- and post-HD conditions (median 34.68 pre-HD versus 34.87 POST; *p* = 0.172).

## 4. Discussion

The present study was focused on the influence of Theranova filter (a medium cut-off membrane) on the immunophenotypic profile of peripheral blood cells. Furthermore, many studies have focused on the comparison between healthy volunteers and HD patients, but only very few have focused on changes after a single HD session in the same individual.

In our study, the percentage of PS-exposing erythrocytes was similar before and after the HD treatment by Theranova filter. In the literature, there is no evidence on the changing of eryptosis induced by a single HD session with a medium cut-off filter. This result is supported by the absence of undesirable effects from the medium cut-off filter on RBCs, and also when considering the composition of the membrane in polyarylethersulfone and polyvinylpyrrolidone blend bPA-free membranes.

One contribution to anemia is the interference of microcirculation caused by the adhesion of eryptotic cells to endothelial cells, which are rapidly cleared from circulating blood.

Excessive eryptosis is a major cause of anemia in ESRD [12]. Here, we have shown that there is no difference before and after the treatment with Therranova filter in terms of eryptosis, thus no worsening of the anemia in ESRD patients occurred.

In a similar manner, Theranova filter did not influence the percentages of lymphocytes (CD3+), lymphocytes T helper (CD3+ and CD4+) cells, and monocytes (CD45+ and CD14+) in a single HD treatment. Based on these results, we hypothesized that Theranova filter contributes to maintaining blood cell homeostasis in HD patients. On the contrary, a reduction in the percentage of blood cells or an immunophenotypic imbalance could be injurious for uremic HD patients.

The expression of HLA-DR is a necessary requirement for antigen recognition and T cell presentation [14]. We observed a significant reduction in the percentage of monocytes expressing HLA-DR after a single Theranova treatment. In addition, we evaluated MFI, which reflects the activation level of cells. In our population, we did not find a change in MFI of HLA-DR. Even if we had reported a lower percentage of monocytes expressing HLA-DR after treatment, these monocytes are more active and reached the same MFI level. We postulated that this was due to a compensatory effect of monocytes trying to preserve the general homeostasis.

In a different context, Steinborn et al. [22] demonstrated that the reduced suppressive activity of Treg cells was not due to a different number of specific types of cells (in that case, Treg cells that can express HLA-DR) but due to their capability to express their protein (in that case HLA-DR). They have also demonstrated that even if the pre-eclamptic patients showed deficiencies in the functional activity of their Treg pool, it contained a high proportion of HLA- DR+ Tregs. Probably, the high proportion of fully developed, highly suppressive DR+ Tregs in these patients compensates for this loss of suppressive activity. In other words, they produce more cells to compensate for less work per cell.

We have reported a similar result in Treg cell populations. We considered CD25+ CD127- as Treg (after selection of CD3+ CD4+ cells). These cells decreased after a single HD treatment with Therranova filter. To better characterize the regulatory capacity of Treg cells, we subdivided Foxp3+ cells (of CD3+ CD4+) on their CD25+ expression. We considered the CD25hi population to be the most specific Treg population.

The FOXP3+ cell population did not change after a single HD treatment with Theranova filter, but the CD3+ CD4+ FOXP3CD25hi population did. There was a significant decrease in this population in accordance with the Treg cells evaluated as CD25CD127- cells.

We found a significantly lower level of T regulatory cells after the HD session with Theranova filter, but we did not observe any changes in the MFI of Foxp3. The MFI of FOXP3 reflects the activity of T regulatory cells. High expressions of FOXP3 correspond to high Treg cell activity [23,24,25,26]. As for changes in HLA-DR levels, we postulated a compensatory effect with less T reg cells that are more active trying to preserve general immune homeostasis. We reported a similar result for CD25. We observed unchanged MFI levels of CD25 in the CD3+ CD4+ FOX3+ CD25hi population, even if this population significantly decrease after the treatment with Therranova filter. Less cells expressed the same global amount of CD25 IL-2 receptor that reflects the Treg state of activation [24,25].

With this pilot observational study, we have revealed an immunomodulation-driven, expanded hemodialysis with a medium cut-off, polyarylethersulfone and polyvinylpyrrolidone blend, BPA-free membrane (Theranova filter), affecting HLA-DR and Treg cells.

This pilot observational study has some limits due to the number of patients. The parameters analyzed were influenced by some of the patients’ characteristics. For example, Tregs are influenced by age, and this is why we did not analyze the absolute number but instead described the changes before and after the HD session. Of course, diabetes and cardiovascular disease can also influence the immunophenotype of patients.

To minimize the influence of each single patient, we described the changes before and after the treatment. This meant that even for patients with different Treg cells numbers before the treatment due to individual differences, we measured the global change after dialysis, thus minimizing the contribution of each single patient. We observed the delta values before and after and not the absolute values.

## 5. Conclusions

Our preliminary results can be considered to be a hypothesis, generating and stimulating further studies with better designs and larger populations.

To the best of our knowledge, the immunophenotypic profile of blood cells in patients that need dialysis through a medium cut-off filter have not been yet investigated. Furthermore, only very few studies have focused on immunophenotypic changes that occur after a single treatment session in the same individual. In conclusion, with this pilot observational study, we have revealed an immunomodulation driven by only one treatment session with Theranova filter. It could be interesting to follow up these patients to verify if the same results are maintained with time. It will be necessary to increase the number of patients and compare different filters to better characterize the immunomodulation led by dialysis and to study different immunomodulation mechanisms driven by different filters with different membrane cut-offs.

## Figures and Tables

**Table 1:** Patient characteristics.

	Patient Characteristics			Treatment			
	Age (at Treatment) 2017	Gender (F/M)	DM (Yes/No)	Kt/V	Qb	UreaPRE	Duration
1	70	M	yes	1.06	248.3	95	4
2	67	M	yes	0.95	210	189.5	4
3	65	M	yes	1.28	350	160	4.5
4	64	M	no	1	213	156.3	4
5	70	M	yes	0.9	200	125	3.5
6	63	F	yes	1.2	300	162	3.5
7	81	M	no	1.25	310	153	4
8	86	M	no	1.3	300	95	3.5
9	84	M	yes	1.2	300	123	3.5
10	71	F	no	1.3	320	159	4
11	79	F	no	0.9	250	121	3.5

**Table 2:** WBC counts and differentiation.

	MedianPRE	MedianPOST	*p* Value
Lymphocytes	51.76; IQR 42.93–60.15	47.7; IQR 41.37–60.81	0.54
T Lymphocytes	18.79; IQR 13.15–23.61	16.95; IQR 8.65–21.81	0.42
Monocytes	18.4; IQR 13.45–31.55	17.1; IQR 11.6–22.85	0.06

## Data Availability

All data generated or analyzed during this study will be included in a future article. Further enquiries can be directed to the corresponding author.
